# Impact of an artificial intelligence‐aided endoscopic diagnosis system on improving endoscopy quality for trainees in colonoscopy: Prospective, randomized, multicenter study

**DOI:** 10.1111/den.14573

**Published:** 2023-05-29

**Authors:** Daisuke Yamaguchi, Ryo Shimoda, Koichi Miyahara, Takahiro Yukimoto, Yasuhisa Sakata, Ayako Takamori, Yumi Mizuta, Yutaro Fujimura, Suma Inoue, Michito Tomonaga, Yuya Ogino, Kohei Eguchi, Kei Ikeda, Yuichiro Tanaka, Hironobu Takedomi, Hidenori Hidaka, Takashi Akutagawa, Nanae Tsuruoka, Takahiro Noda, Seiji Tsunada, Motohiro Esaki

**Affiliations:** ^1^ Department of Gastroenterology National Hospital Organization Ureshino Medical Center Ureshino Japan; ^2^ Division of Gastroenterology, Department of Internal Medicine, Faculty of Medicine Saga University Saga Japan; ^3^ Department of Endoscopic Diagnostics and Therapeutics Saga University Hospital Saga Japan; ^4^ Clinical Research Center Saga University Hospital Saga Japan; ^5^ Department of Internal Medicine Karatsu Red Cross Hospital Saga Japan

**Keywords:** adenoma detection rate, adenoma miss rate, colonoscopy, computer‐aided diagnosis, trainee

## Abstract

**Objective:**

This study was performed to evaluate whether the use of CAD EYE (Fujifilm, Tokyo, Japan) for colonoscopy improves colonoscopy quality in gastroenterology trainees.

**Methods:**

The patients in this multicenter randomized controlled trial were divided into Group A (observation using CAD EYE) and Group B (standard observation). Six trainees performed colonoscopies using a back‐to‐back method in pairs with gastroenterology experts. The primary end‐point was the trainees' adenoma detection rate (ADR), and the secondary end‐points were the trainees' adenoma miss rate (AMR) and Assessment of Competency in Endoscopy (ACE) tool scores. Each trainee's learning curve was evaluated using a cumulative sum (CUSUM) control chart.

**Results:**

We analyzed data for 231 patients (Group A, *n* = 113; Group B, *n* = 118). The ADR was not significantly different between the two groups. Group A had a significantly lower AMR (25.6% vs. 38.6%, *P* = 0.033) and number of missed adenomas per patient (0.5 vs. 0.9, *P* = 0.004) than Group B. Group A also had significantly higher ACE tool scores for pathology identification (2.26 vs. 2.07, *P* = 0.030) and interpretation and identification of pathology location (2.18 vs. 2.00, *P* = 0.038). For the CUSUM learning curve, Group A showed a trend toward a lower number of cases of missed multiple adenomas by the six trainees.

**Conclusion:**

CAD EYE did not improve ADR but decreased the AMR and improved the ability to accurately locate and identify colorectal adenomas. CAD EYE can be assumed to be beneficial for improving colonoscopy quality in gastroenterology trainees.

**Trial registration:**

University Hospital Medical Information Network Clinical Trials Registry (UMIN000044031).

## INTRODUCTION

Colonoscopy is the gold standard for diagnosing colorectal diseases, and its implementation contributes to decreasing mortality in patients with colorectal cancer.^1^, ^2^, ^3^ However, the quality of colonoscopy depends on the endoscopist's proficiency; high‐quality endoscopy is characterized by a high adenoma detection rate (ADR) with few missed adenomas.^4^ Various image‐enhanced endoscopy systems can improve the detectability of colorectal adenomas and reduce the adenoma miss rate (AMR), thereby significantly improving the quality of colonoscopy examinations.^5^, ^6^, ^7^

The application of artificial intelligence (AI) in colonoscopy has attracted attention because of its potential to improve the quality of colonoscopy.^8^ Several computer‐aided diagnosis (CAD) systems have been developed, and their efficacy has been revealed in prospective studies.^9^

CAD EYE (Fujifilm, Tokyo, Japan) has been newly approved and licensed as a medical device using AI technology.^10^ The CAD EYE system enables rapid detection of colorectal polyps, potentially reducing the number of missed adenomas, and its usefulness has been reported in several articles.^11^, ^12^, ^13^, ^14^, ^15^

Developing trainees into competent endoscopists is a central objective of gastroenterology fellowship training. Quality indicators such as the ADR are often used to evaluate training methods and perform regular inspections.^16^, ^17^ Gastroenterology trainees must improve the quality of colonoscopy by increasing the ADR and strive to become full‐fledged endoscopists. Various training methods are available for this purpose.^18^, ^19^, ^20^

We designed this study based on the hypothesis that gastroenterology trainees' use of CAD EYE in colonoscopy improves the ADR and decreases the AMR compared with standard colonoscopy.

## METHODS

### Study population and ethics

This prospective, multicenter, randomized trial involved adult patients scheduled for elective colonoscopy. The study was conducted at three tertiary medical centers: Ureshino Medical Center, Karatsu Red Cross Hospital, and Saga University Hospital. It was performed in accordance with the Declaration of Helsinki and reported following the Consolidated Standards of Reporting Trials (CONSORT) guidelines. The study protocol and consent procedure were approved by the Ethics Review Committee of the National Hospital Organization Ureshino Medical Center (approval number 20‐94), and all patients provided informed consent. The trial was registered with the University Hospital Medical Information Network Clinical Trials Registry (UMIN000044031) on 26 April 2021.

The study involved adults aged ≥20 years who were scheduled for outpatient colonoscopy from May 2021 to March 2022. The colonoscopy examinations were performed either because of a positive fecal immunochemical test or for surveillance after colonic polypectomy. We excluded patients with: ileus, suspected bowel obstruction, or toxic megacolon; prior abdominal or pelvic surgery; inflammatory bowel disease; advanced malignancy; severe liver damage (Child–Pugh grade C); dementia or other cognitive disorders; hypersensitivity to bowel preparation drugs for colonoscopy; and pregnancy or lactation.

**Figure 1:**
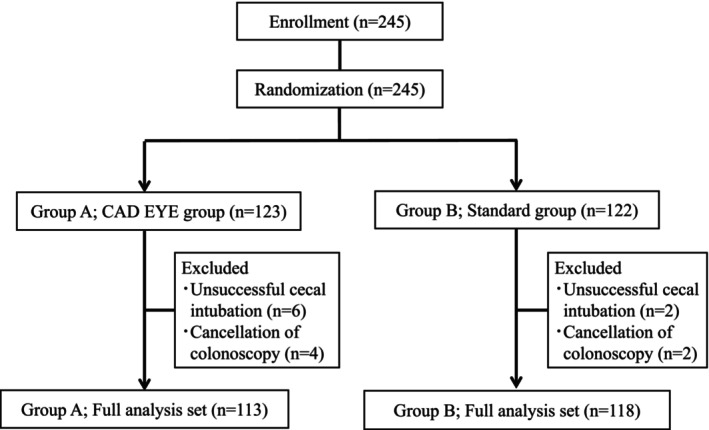
Study flowchart of patient randomization and inclusion in the analyzed groups.

Patients who met the eligibility criteria were sequentially allocated into two groups using an Internet‐based random number generator (Fig. 1). Concealment of randomization was retained by personnel who were not involved in the colonoscopy procedure, the outpatient department, or data collection and analysis.

### Study design

The patients were randomly assigned to Group A (colonoscopic observation with white‐light imaging using CAD EYE) or Group B (standard observation without CAD EYE). The details of the CAD EYE system are described in Appendix S1 and Figure S1. In both groups, six gastroenterology trainees (endoscopists in their third or fourth year as a physician) with limited colonoscopy experience (0–20 cases) who had started their fellowship training and performed all colonoscopies in pairs with a gastroenterology expert (experience with >5000 cases). All colonoscopies were performed with an EC‐L600ZP7 (Fujifilm).

In both groups, the colonoscope was inserted without CAD EYE. The trainees performed colonoscopies in accordance with a back‐to‐back method in pairs with the experts (Appendix S2, Fig. S2). During the back‐to‐back method, the trainee first observed and measured the polyps; the expert was not present for this examination and observed the same area again without prior information. In Group A, computer‐aided characterization (CADx) was used to diagnose lesions if necessary when measuring polyps. The final diagnosis was adopted if the endoscopist changed the diagnosis with CAD EYE during the observation, as previously described.^12^ Lesions were resected endoscopically when possible, and the ADR was calculated for pathologically confirmed adenomas.

After each examination, the expert calculated the ADR, polyp detection rate (PDR), and AMR. The expert also calculated the Assessment of Competency in Endoscopy (ACE) tool scores and measured learning curves for missed adenomas. No study outcomes were made available to the trainees until the end of the study.

Patient demographics, endoscopist information, indication for colonoscopy, cecal intubation time, withdrawal time, lesion information, PDR, ADR, AMR, modified Aronchick Bowel Preparation Scale score, and Boston Bowel Preparation Scale score were recorded for each colonoscopy. Withdrawal time was defined as the sum of the trainee's endoscopic observation time of the four segments. The time spent for polypectomy and magnifying observations was excluded from the withdrawal time. Perioperative severe adverse events were also recorded (perforations, bleeding, diverticulitis, cardiovascular events, severe abdominal pain, or death).

### Study outcomes

The primary outcome was the trainee's ADR. The secondary outcomes were the trainee's AMR and ACE tool scores. We also calculated the PDR, mean number of polyps per patient (MPP), mean number of adenomas per patient (MAP), and cumulative sum (CUSUM) learning curves. The ADR and PDR were defined as the percentage of all examinations that detected one or more adenomas or polyps. Based on the hypothesis that experts miss fewer adenomas than trainees, the AMR was defined as follows: (number of adenomas found by experts − number of adenomas found by trainees)/(number of adenomas found by experts) × 100.

In 2014, the Training Committee of the American Society for Gastrointestinal Endoscopy developed the ACE tool.^17^ In this study we employed the ACE tool as a more formalized and objective training assessment method for trainees. The details of the ACE tool are described in Appendix S3.

### Sample size calculation and statistical analysis

We calculated the sample size based on previous studies^21^, ^22^, ^23^ and on the trend reports from the colonoscopy data in our hospital from November 2019 to November 2020. Previous studies have suggested an ADR of 27.0% to 35.0% in a gastroenterology trainee's first year. We expected a 20% increase in the ADR in Group A over Group B to be a minimal clinically important difference; thus, 91 patients per group were required with a significance level of 5% and power level of 80%. Additionally, assuming that 20% of patients had no polyps, 110 patients were required per group. The target total number of patients was 240 (120 each in both groups), considering discontinuation and dropout.

Categorical data are expressed as number (percentage), and the χ^2^‐test was used to investigate differences between the two groups. Numerical data are expressed as mean ± standard deviation, and Student's *t*‐test was used to determine differences between the two groups. We treated the ACE tool score as an interval variable with equally spaced progression of competency along the scoring scale, and we compared ACE tool scores using *t*‐tests.

CUSUM learning curve analyses were conducted in both groups to clarify trends and visualize changes in the multiple AMR for the six trainees.^24^, ^25^, ^26^, ^27^, ^28^, ^29^ The details of the CUSUM learning curve analyses are described in Appendix S4 and Figure S3. The CUSUM learning curve was drawn by plotting the performance score on the *y*‐axis and the procedure number on the *x*‐axis. The performance score decreased by 0.293 with each success and increased by 0.707 with each failure. Success was defined as the trainee not missing multiple adenomas, and failure was defined as the trainee missing multiple adenomas.

A *P*‐value of <0.05 indicated statistical significance, and all statistical analyses were performed with JMP v. 16.0.0 (SAS Institute, Cary, NC, USA).

## RESULTS

### Baseline characteristics

Figure 1 shows the patient flowchart. In total, 245 patients were enrolled and randomized to Group A (*n* = 123) or Group B (*n* = 122). Eight patients (Group A, *n* = 6; Group B, *n* = 2) who underwent unsuccessful cecal intubation and six patients (Group A, *n* = 4; Group B, *n* = 2) whose colonoscopy was canceled for the patients' convenience were excluded. Thus, 231 patients completed the study protocol and were included in the final analysis set (Group A, *n* = 113; Group B, *n* = 118).

Table 1 compares the baseline characteristics between the two groups. The most common indication for examination was a positive fecal immunochemical test. None of the patient‐related factors differed between the two groups. In addition, there were no significant differences in the use of sedatives and analgesics or the degree of bowel cleansing (modified Aronchick Bowel Preparation Scale score and Boston Bowel Preparation Scale score).

**Table 1:** Patients' baseline characteristics

	Group A	Group B	*P*‐value
Number of patients (*N*)	113	118	–
Age (years)	63.1 ± 10.8	63.3 ± 11.8	0.907
Sex, male	58 (51.3)	66 (55.9)	0.511
BMI (%)	23.6 ± 3.5	23.6 ± 4.5	0.980
Family history of CRC	13 (11.5)	13 (11.0)	1.000
Comorbidity			
Hypertension	29 (25.7)	24 (20.3)	0.352
Malignant diseases	11 (9.7)	18 (15.3)	0.237
Diabetes mellitus	16 (14.2)	10 (8.5)	0.213
Cardiovascular diseases	6 (5.3)	7 (5.9)	1.000
Chronic kidney diseases	3 (2.7)	4 (3.4)	1.000
Cerebrovascular diseases	3 (2.7)	3 (2.5)	1.000
Indication for examination			
FIT positive	53 (46.9)	64 (54.2)	0.294
Detailed examination	22 (19.5)	26 (22.0)	0.746
Polyp surveillance	15 (13.3)	13 (11.0)	0.688
Any abdominal symptoms	13 (11.5)	10 (8.5)	0.513
Others	10 (8.9)	5 (4.2)	0.187
Using sedative agent	60 (53.1)	72 (61.2)	0.234
Using analgesic agent	13 (11.5)	15 (12.7)	0.842
Modified Aronchick Scale			
Excellent	77 (68.1)	68 (57.6)	0.104
Good	21 (18.6)	31 (26.3)	0.207
Fair	13 (11.5)	14 (11.9)	1.000
Poor/inadequate	2 (1.8)	5 (4.2)	0.447
Boston Bowel Preparation Score			
Whole colon	8.4 ± 1.2	8.2 ± 1.5	0.272

Results are presented as mean ± standard deviation, *n*, or *n* (%).

BMI, body mass index; CRC, colorectal cancer; FIT, fecal immunochemical test.

### ADR, PDR, and AMR

Table 2 compares the quality indices of colonoscopy including ADR, PDR, and AMR between Group A and Group B.

**Table 2:** Adenoma detection rate (ADR), polyp detection rate (PDR), and adenoma miss rate (AMR)

	Group A	Group B	*P*‐value
Number of patients (*N*)	113	118	–
Number of patients with adenomas	66	72	–
ADR (%)	58.4	61.0	0.690
Number of adenomas	157	167	–
Mean number of adenomas per patient	1.4 ± 1.8	1.4 ± 2.0	0.945
Number of patients with polyps	69	74	–
PDR (%)	61.1	62.7	0.892
Number of polyps	169	215	–
Mean number of polyps per patient	1.5 ± 1.9	1.8 ± 2.3	0.241
Total number of adenomas detected by experts	211	272	–
Number of missed adenomas	54	105	–
AMR (%)	25.6	38.6	0.033
Mean number of missed adenomas per patient	0.5 ± 0.8	0.9 ± 1.2	0.004

Results are presented as mean ± standard deviation or *n* unless otherwise indicated.

The trainees' ADR was 58.4% in Group A and 61.0% in Group B, with no significant difference (*P* = 0.690). Group A had a significantly lower AMR (25.6% vs. 38.6%, *P* = 0.033) and mean number of missed adenomas per patient (0.5 vs. 0.9, *P* = 0.004) than Group B. The trainees' PDR was 61.1% for Group A and 62.7% for Group B, with no significant difference (*P* = 0.892). The MAP and MPP were also similar between the two groups.

### Clinicopathologic features of detected polyps

Table 3 summarizes the clinicopathologic features of polyps detected in the two groups. Regarding location, the polyps were most commonly located in descending order of the ascending colon, sigmoidal colon, and transverse colon. In terms of morphology, 0‐Is polyps and polyps of ≤5 mm were more common. There were no significant differences in the polyp characteristics between the two groups. Pathologically, most patients had adenoma with low‐grade dysplasia, and one invasive cancer was found in Group A and three in Group B. The ADR was not significantly different between the two groups.

**Table 3:** Clinicopathologic features of detected polyps

	Group A		Group B		*P*‐value
	*n*	Number of polyps per patient	*n*	Number of polyps per patient	
Location					
Cecum	17	0.15 ± 0.45	19	0.16 ± 0.47	0.608
Ascending colon	60	0.53 ± 0.90	83	0.70 ± 1.30	0.771
Transverse colon	56	0.50 ± 0.77	72	0.70 ± 1.45	0.920
Descending colon	38	0.34 ± 0.73	52	0.44 ± 0.77	0.908
Sigmoidal colon	63	0.55 ± 0.82	79	0.67 ± 0.95	0.770
Rectum	15	0.13 ± 0.39	23	0.19 ± 0.51	0.736
Morphology^†^					
0‐Is	197	1.74 ± 2.17	261	2.21 ± 2.75	0.918
0‐Isp	12	0.11 ± 0.39	18	0.15 ± 0.58	0.850
0‐Ip	4	0.04 ± 0.19	7	0.06 ± 0.27	0.765
0‐IIa	35	0.31 ± 0.86	38	0.32 ± 1.07	0.380
0‐IIb	1	0.01 ± 0.09	0	0.00 ± 0.00	0.432
0‐IIc, 0‐IIa + IIc	0	0.00 ± 0.00	4	0.03 ± 0.18	0.138
Size					
<5 mm	153	1.35 ± 1.68	188	1.59 ± 2.06	0.347
5–10 mm	86	0.76 ± 1.23	120	1.02 ± 1.53	0.661
>10 mm	10	0.09 ± 0.32	20	0.17 ± 0.53	0.344
Histopathology					
Hyperplastic polyp	27	0.24 ± 0.63	39	0.33 ± 0.65	0.792
Sessile serrated adenoma/polyp	1	0.01 ± 0.09	4	0.03 ± 0.22	0.396
Traditional serrated adenoma	0	0.00 ± 0.00	2	0.02 ± 0.13	0.509
Adenoma with low‐grade dysplasia	200	1.76 ± 1.96	252	2.14 ± 2.69	0.359
Adenoma with high‐grade dysplasia	9	0.09 ± 0.34	11	0.09 ± 0.32	1.000
Invasive cancer	1	0.01 ± 0.09	3	0.03 ± 0.16	0.638
Other	11	0.10 ± 0.30	17	0.14 ± 0.44	0.701

^†^ Morphology is described according to the Paris endoscopic classification.

Results are presented as mean ± standard deviation or *n*.

Table S1 shows the clinicopathologic characteristics of the missed adenomas in both groups. Most of the missed adenomas were 0‐Is, ≤5 mm, and located in the ascending and transverse colon.

The mean withdrawal time of each procedure in Group A and B was 13.06 ± 3.23 and 13.05 ± 4.01 min, respectively, with no significant difference (*P* = 0.977) (Table S2).

### ACE tool scores

Table 4 summarizes the ACE tool evaluation. Group A scored significantly higher than Group B on two items for competency in cognitive skills: pathology identification (2.26 vs. 2.07, *P* = 0.030) and interpretation and identification of pathology location (2.18 vs. 2.00, *P* = 0.038). The two groups did not significantly differ in the remaining five items of the cognitive skills score. All seven items of the motor skill scores were not significantly different between the two groups.

**Table 4:** Evaluation by Assessment of Competency in Endoscopy tool scores

	Group A	Group B	*P*‐value
Number of patients (*N*)	113	118	
Motor skills			
Effective use of air, water, and suction	2.21 ± 0.83	2.13 ± 0.78	0.429
Scope steering technique	2.09 ± 0.84	2.10 ± 0.79	0.902
Fine tip control	2.03 ± 0.85	1.89 ± 0.82	0.216
Loop reduction techniques	2.09 ± 0.88	2.01 ± 0.83	0.479
Visualization of mucosa	2.32 ± 0.85	2.15 ± 0.78	0.105
Ability to apply therapeutic tools	2.27 ± 0.71	2.16 ± 0.72	0.232
Overall hands‐on competence	2.18 ± 0.85	2.13 ± 0.84	0.654
Cognitive skills			
Lumen identification	2.24 ± 0.83	2.18 ± 0.80	0.559
Knowledge of indication and medical issues	2.32 ± 0.74	2.23 ± 0.71	0.346
Management of patient discomfort	2.38 ± 0.78	2.25 ± 0.71	0.200
Pathology identification and interpretation	2.26 ± 0.78	2.07 ± 0.72	0.030
Identifying location of pathology	2.18 ± 0.80	2.00 ± 0.78	0.038
Knowledge of therapeutic tool	2.28 ± 0.82	2.19 ± 0.85	0.378
Overall cognitive competence	2.30 ± 0.78	2.18 ± 0.78	0.232

Results are presented as mean ± standard deviation. Scoring was based on a 4‐point scale (1, novice; 2, intermediate; 3, advanced; 4, superior).

### CUSUM learning curves

**Figure 2:**
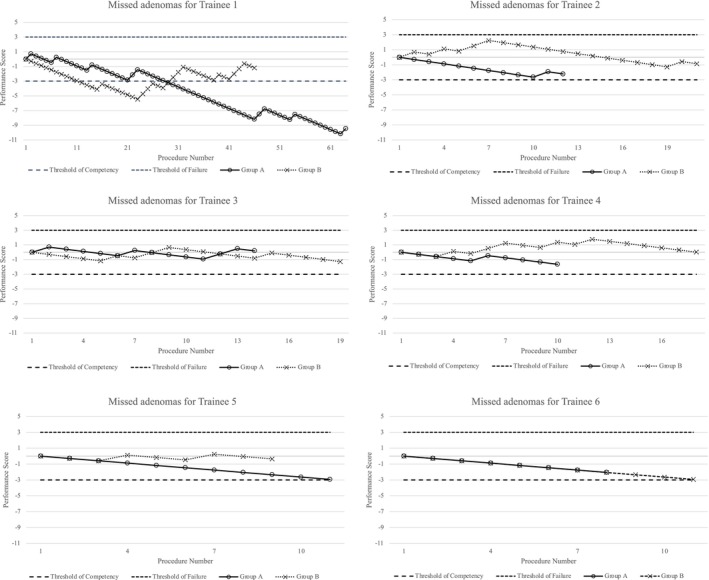
Cumulative sum learning curves for the six trainees.

Figure 2 shows CUSUM learning curves for the two groups. Group A showed a trend toward a lower number of cases in which multiple adenomas were missed by the six trainees who performed colonoscopies. Even after accumulating cases, the number of missed adenomas was consistently lower in Group A. After accumulating more than 10 cases, the number of missed adenomas tended to gradually increase in Group B.

## DISCUSSION

Recent randomized trials have demonstrated the effectiveness of AI in patients undergoing screening colonoscopy, and computer‐aided detection (CADe) systems have been shown to increase the ADR as the main quality indicator of colonoscopy.^9^, ^30^, ^31^ In one study, the expert's accuracy, sensitivity, and specificity were not statistically different from those of CADx alone when characterized with blue laser imaging, and CADx was more accurate than the expert when characterized with white‐light imaging.^10^

A unique aspect of this multicenter randomized controlled trial was the examination of the usefulness of colonoscopy with CAD EYE, which includes both the CADe and CADx systems, for colonoscopy trainees. In six trainees with less colonoscopy experience, the ADR, MAP, PDR, and MPP were not different between the CAD EYE group and the standard observation group. The ADR of trainees in this study was unexpected because it was >20% higher than the ADRs in previous reports.^21^, ^22^, ^23^ Thus, there may have been no significant difference between the CAD EYE group and the standard observation group. A possible explanation for such trainees' high ADR could be as follows. First, because this study used the back‐to‐back method when observing each colorectal section, a more favorable condition regarding the amount of air and intestinal fluid could be created for the endoscopic observation by the trainees. Second, longer withdrawal time could be the result of more careful endoscopic observation to avoid experts' detection of missed polyps by trainees.

Notably, however, the trainees' AMR and number of missed adenomas per patient were significantly lower in the CAD EYE group. Inconsistent results between ADR and AMR could be mainly attributed to the unblinded nature of the present study. Namely, the condition of endoscopic observation was different from that of daily clinical practice, which could influence the detectability of adenomas by the trainees. However, the diagnostic ability of adenomas without CAD EYE is mainly based on the knowledge and experience of colonic pathologies and is less influenced by the condition of endoscopic observation. In addition, considering that the results regarding missed polyps in patients with multiple polyps are not reflected in ADR, it seems inevitable to assess AMR for the evaluation of the clinical impact of CAD EYE.

There was no difference in the ACE tool score for motor skills with or without CAD EYE, but two cognitive scores of the ACE tool were significantly better in the CAD EYE group: pathology identification and interpretation and identification of pathology location. These results suggest that the CADx system of CAD EYE may have assisted in the trainees' diagnosis of adenomas. Image‐enhanced endoscopy‐magnified observations have high accuracy for the differential diagnosis between neoplastic and hyperplastic lesions.^32^, ^33^ Still, magnifying observation and clinical judgment based on narrow‐band imaging findings require extensive experience and take a longer time to master than standard nonmagnifying observation, which is independent of proficiency and experience. During the developmental process of the CADx system in CAD EYE, endoscopists used both magnifying and nonmagnifying images as learning images; therefore, it can be performed under both conditions.^13^ Hence, this CADx system might effectively adapt to learn magnifying observation and the diagnosis of adenomas, which would be helpful for less experienced endoscopists.

In this study, ACE tool scores and CUSUM learning curves were used as objective indices in colonoscopy performed by trainees. Global skill assessment in colonoscopy is complicated and ambiguous. In a study of trainees, the problem was even more acute because of large differences in the number of trainees, consistency of training sites, length of evaluation time, threshold for attending physician assistance, and knowledge of objective assessment items.^17^, ^20^ In Japan, there is still no general indicator to evaluate trainees' endoscopic skills; however, the ACE tool score seems to have potential. Furthermore, the CUSUM learning curve in this study showed that continued use of CAD EYE tended to improve performance to avoid missing multiple adenomas. Continually monitoring the CUSUM learning curve may provide additional insight into colonoscopy training attainment.^7^ As more cases are accumulated in the future, the point to be reached will become more apparent. The combination of established quality indicators such as the ADR and comprehensive technology and exercise assessment, including the ACE tool and real‐time monitoring of CUSUM learning curves, is considered a validated evaluation method for the colonoscopic evaluation of trainees.

The present study has several limitations. First, the proportion of patients with colorectal polyps was slightly lower than expected. Thus, the actual number of patients enrolled in the present study could be statistically underpowered. Second, the actual number of polyps was defined as the number detected by the expert. Therefore, the ADR and PDR might have been calculated to be higher if the expert missed polyps. In contrast, some lesions could have been missed by CAD EYE, and misrecognition was common, especially in the cecum and rectum. CADe is expected to improve the ability to recognize difficult‐to‐detect lesions in the future. Third, because of the nature of CAD EYE, neither the trainee nor the specialist could be blinded to the use of CAD EYE, and the ACE tool scores may have been subject to bias by the judges. Fourth, the number of colonoscopies varied among six trainees because of the different circumstances at each institution, which might affect the result of the CUSUM learning curves. Prospective studies should be conducted to increase the number of trainees and standardize the number of cases.

In conclusion, CAD EYE did not change trainees' ADR but decreased trainees' AMR and improved the ability to accurately detect and identify colorectal adenomas. Thus, CAD EYE is considered to be beneficial for colonoscopy in beginning endoscopists.

## CONFLICT OF INTEREST

Authors declare no conflict of interest for this article.

## FUNDING INFORMATION

This study was supported by an academic research grant from the Japan Gastroenterological Endoscopy Society in 2021.

## Supporting information


**Appendix S1** Description of the CAD EYE system (Fujifilm, Tokyo, Japan).
**Appendix S2** Colonoscopic observation by back‐to‐back method in both groups.
**Appendix S3** Assessment of Competency in Endoscopy (ACE) tool.
**Appendix S4** Cumulative sum (CUSUM) learning curve analysis.

**Figure S1** Endoscopic figures of CAD EYE (Fujifilm, Tokyo, Japan).
**Figure S2** Colonoscopic observation by back‐to‐back method.
**Figure S3** Equations and constants for cumulative sum (CUSUM) analysis.

**Table S1** Clinicopathologic features of missed adenomas.
**Table S2** Withdrawal time of each procedure.

## Data Availability

The datasets used and/or analyzed during the current study are available from the corresponding author on reasonable request.
