# Supplementary material for: Impact of an artificial intelligence‐aided endoscopic diagnosis system on improving endoscopy quality for trainees in colonoscopy: Prospective, randomized, multicenter study
Source: Dig Endosc. 2023 May 29;36(1):40–8. doi: 10.1111/den.14573 (PMC12136242; doi:10.1111/den.14573)
Supplement: Supplementary file 1 — Appendix S1 Description of the CAD EYE system (Fujifilm, Tokyo, Japan). Appendix S2 Colonoscopic observation by back‐to‐back method in both groups. Appendix S3 Assessment of Competency in Endoscopy (ACE) tool. Appendix S4 Cumulative sum (CUSUM) learning curve analysis. [file DEN-36-40-s002.docx]

**Supplementary material**

Appendix S1. Description of CAD EYE system

The CAD EYE (Fujifilm, Tokyo, Japan) is a new AI system developed to detect colorectal polyps.^11^ The endoscopist activates the CAD EYE by pushing a button on the handle of the endoscope. For CADe, the annotation box surrounds a polyp when CAD EYE detects a polyp with white-light imaging. An alarm then sounds to draw attention to the polyp when the polyp can be recognized with the annotation box for about 0.2 to 0.3 s.^10^ In this study, the recognition of a polyp with CAD EYE was defined when the alarm rang. For CADx, the diagnosis of two categories (“NEOPLASTIC” or “HYPERPLASTIC”) on BLI (Blue LASER/Light Imaging) with CAD EYE is shown at the bottom of the monitor when a polyp is recognized. Simultaneously, yellow or green curved lines, signaling “neoplastic” and “hyperplastic” lesions, respectively, are shown around the endoscopic monitor in the lower right window to indicate that CAD EYE has detected a polyp. In this study, the diagnostic capability of CAD EYE was defined when the endoscopist showed the diagnosis at the bottom of the monitor (>1 s).

Appendix S2. Colonoscopic observation by back-to-back method in both groups

If the trainee could not advance the endoscope to the cecum within 15 minutes, the expert took turns with the trainee and inserted the scope to avoid burdening the patient, then switched to the trainee when the endoscope reached to the cecum before starting the observation. Sedatives and analgesics were used at the endoscopist’s discretion. To avoid burdening the patient, the entire colon was divided into four segments: (1) ascending colon: from the cecum to the hepatic flexure, (2) transverse colon: from the hepatic flexure to the splenic flexure, (3) descending colon: from the splenic flexure to the SD junction, and (4) sigmoid colon and rectum: from the SD junction to the anus. Each segment was observed using the back-to-back method by a first-time trainee and a second-time expert in turn (Supplementary Fig. 1). The expert was defined as those with at least 5,000 colonoscopies, and ten experts were involved in this study, similar to the two groups.

In both groups, checkpoints were set up between (1) and (2), between (2) and (3), between (3) and (4), and after (4). After the trainee found polyps and adenomas, the expert confirmed the polyps and adenomas and performed additional observation and endoscopic polypectomy or endoscopic mucosal resection if necessary.

Appendix S3. The ACE tool

The ACE tool consists of 14 questions measuring individual cognitive and motor skills in colonoscopy and 2 questions assessing overall cognitive and motor skills.^20^ These questions are scored on a 4-point scale (1=novice, 2=intermediate, 3=advanced, 4=superior). A score of 4 indicates competency in that specific skill or that broad area. Two additional questions in the ACE tool (independent polyp detection and maximum extent of intubation) are outside the 4-point scale. They are designed to calculate polyp detection and the independent cecal intubation rate. We assessed independent polyp detection as the PDR for these two items. The maximum extent of intubation was excluded from the assessment because it is not associated with evaluation by CAD EYE.

Appendix S4. CUSUM learning curve analysis

CUSUM was initially developed for industrial quality management and allowed easy detection of trends toward predefined success or failure objectives.^24^ In healthcare, it has been adapted to assess competency and build learning curves for training in several endoscopic and surgical procedures.^19,25-28^

CUSUM learning curves are built with a performance score initially at a baseline of 0 that is updated with each iteration of the procedure in question. If the procedure meets the predefined quality measure, an amount *s* is subtracted from the score. If the procedure fails to meet that measure, *1−s* is added to the score. The constant *s* is derived from an intrinsic failure rate (p0) and an unacceptable failure rate (p1) of the metric in question. In this study, we assumed that the probability of missing multiple adenomas was 20% for competent gastroenterologist experts.^5,10^ Therefore, we set p0=0.2. Consistent with typical practice, we set p1=0.4, twice the value of p0.^24^ Given our assumptions for p0 and p1, we determined that s=0.293. Therefore, the performance score would decrease by 0.293 with each success and increase by 0.707 with each failure.

A CUSUM learning curve is drawn by plotting the performance score on the y-axis versus the attempt number on the x-axis. The trend toward competency is established by a horizontal or negative deflection of the curve, whereas the opposite trend is established by an upward deflection of the curve. Competency is reached when the curve crosses the threshold of competency (H1) from above. Per convention,^29^ we assumed that the probability for type 1 errors was 0.05 and that of type 2 errors was 0.2 and, given the above assumptions, determined that H1 and H0 were approximately 3. Further details on the calculation of CUSUM parameters are available in the supplementary materials^18^ (Supplementary Fig. 2).
