# Supplementary material for: Impact of an artificial intelligence‐aided endoscopic diagnosis system on improving endoscopy quality for trainees in colonoscopy: Prospective, randomized, multicenter study
Source: Dig Endosc. 2023 May 29;36(1):40–8. doi: 10.1111/den.14573 (PMC12136242; doi:10.1111/den.14573)
Supplement: Supplementary file 2 — Figure S1 Endoscopic figures of CAD EYE (Fujifilm, Tokyo, Japan). Figure S2 Colonoscopic observation by back‐to‐back method. Figure S3 Equations and constants for cumulative sum (CUSUM) analysis. [file DEN-36-40-s003.pdf]

# Supplementary Figure 1

CADe | CADx “HYPERPLASTIC” | CADx “NEOPLASTIC”

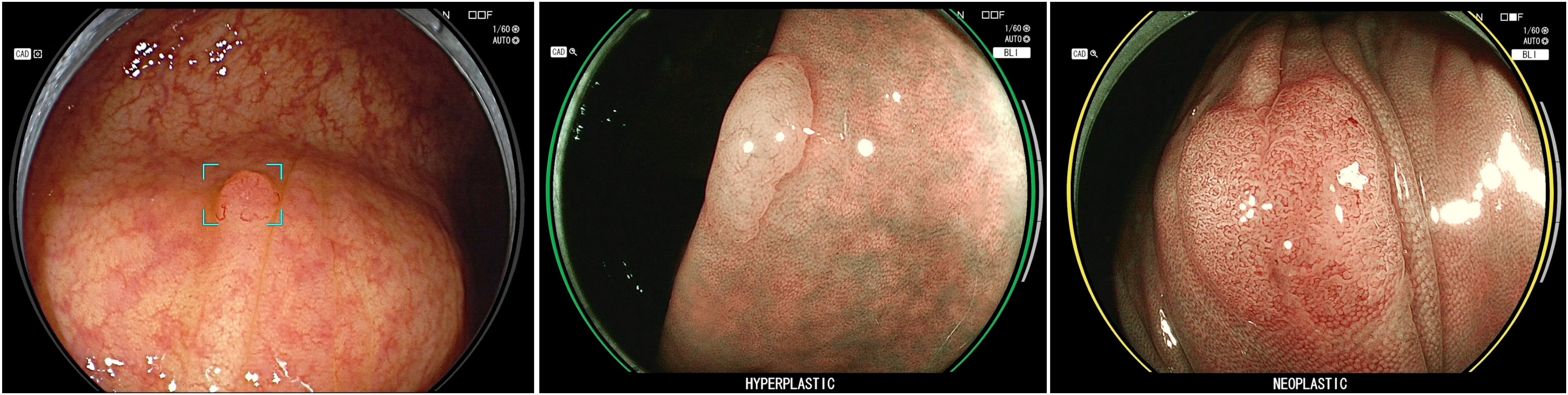

## Supplementary Figure 2

**Group A**

① Ascending colon
Observation with CAD EYE
check point
↓
② Transverse colon
Observation with CAD EYE
check point
↓
③ Descending colon
Observation with CAD EYE
check point
↓
④ Sigmoidal colon-Rectum
Observation with CAD EYE
check point

**Group B**

① Ascending colon
Observation without CAD EYE
check point
↓
② Transverse colon
Observation without CAD EYE
check point
↓
③ Descending colon
Observation without CAD EYE
check point
↓
④ Sigmoidal colon-Rectum
Observation without CAD EYE
check point

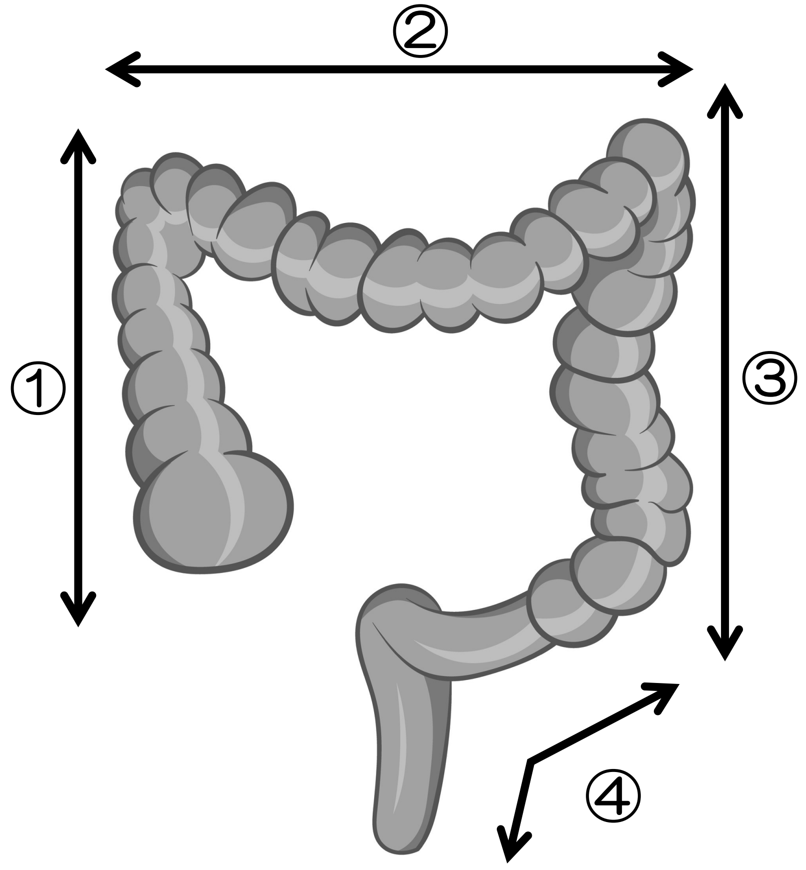

## Supplementary Figure 3

$p_0$ (acceptable or intrinsic failure rate) = 0.20
$p_1$ (unacceptable failure rate) = 0.40
$\alpha$ (type I failure) = 0.05
$\beta$ (type II failure) = 0.20

$$a = \ln\left(\frac{1-\beta}{\alpha}\right)$$

$$P = \ln\left(\frac{p_1}{p_0}\right)$$

$$Q = \ln\left(\frac{1-p_0}{1-p_1}\right)$$

$$s = \frac{Q}{(P+Q)}$$

$$H_1 = -\frac{a}{P+Q}$$

$$H_0 = \frac{a}{P+Q}$$
