# Supplementary material for: Impact of an artificial intelligence‐aided endoscopic diagnosis system on improving endoscopy quality for trainees in colonoscopy: Prospective, randomized, multicenter study
Source: Dig Endosc. 2023 May 29;36(1):40–8. doi: 10.1111/den.14573 (PMC12136242; doi:10.1111/den.14573)
Supplement: Supplementary file 3 — Table S1 Clinicopathologic features of missed adenomas. Table S2 Withdrawal time of each procedure. [file DEN-36-40-s001.docx]

Supplementary Table 1. Clinicopathologic features of missed adenomas

|  | Group A | | Group B | |  |
| --- | --- | --- | --- | --- | --- |
|  | n | Number of polyps per patient | n | Number of polyps per patient | *P* value |
| Location |  |  |  |  |  |
| Cecum | 5 | 0.09 ± 0.29 | 4 | 0.04 ± 0.19 | 0.220 |
| Ascending colon | 15 | 0.28 ± 0.45 | 37 | 0.35 ± 0.48 | 0.337 |
| Transverse colon | 14 | 0.26 ± 0.44 | 25 | 0.24 ± 0.43 | 0.773 |
| Descending colon | 9 | 0.17 ± 0.38 | 14 | 0.13 ± 0.34 | 0.587 |
| Sigmoidal colon | 9 | 0.17 ± 0.38 | 23 | 0.22 ± 0.42 | 0.424 |
| Rectum | 2 | 0.04 ± 0.19 | 2 | 0.02 ± 0.14 | 0.540 |
| Morphology |  |  |  |  |  |
| 0-Is | 45 | 0.83 ± 0.38 | 87 | 0.83 ± 0.38 | 0.940 |
| 0-Isp | 2 | 0.04 ± 0.19 | 1 | 0.01 ± 0.10 | 0.323 |
| 0-Ip | 0 | 0.00 ± 0.00 | 2 | 0.02 ± 0.14 | 0.158 |
| 0-IIa | 7 | 0.31 ± 0.86 | 15 | 0.32 ± 1.07 | 1.000 |
| Size |  |  |  |  |  |
| <5 mm | 43 | 0.80 ± 0.41 | 87 | 0.83 ± 0.34 | 0.629 |
| 5–10mm | 11 | 0.20 ± 0.41 | 16 | 0.15 ± 0.36 | 0.436 |
| >10 mm | 0 | 0.00 ± 0.00 | 2 | 0.02 ± 0.14 | 0.158 |
| Histopathology |  |  |  |  |  |
| Adenoma with low-grade dysplasia | 54 | 1.00 ± 0.00 | 104 | 0.99 ± 0.10 | 0.320 |
| Adenoma with high-grade dysplasia | 0 | 0.00 ± 0.00 | 1 | 0.01 ± 0.01 | 0.320 |

Results are presented as mean ± standard deviation or n.

*Morphology is described according to the Paris endoscopic classification.

Supplementary Table 2. Withdrawal time of each procedure

|  | Group A | Group B | *P* value |
| --- | --- | --- | --- |
| Number of patients (N) | 113 | 118 |  |
| Total | 13.06 ± 3.23 | 13.05 ± 4.01 | 0.977 |
| Ascending colon | 3.13 ± 1.28 | 3.31 ± 2.03 | 0.374 |
| Transverse colon | 2.38 ± 1.31 | 2.59 ± 1.33 | 0.232 |
| Descending colon | 2.13 ± 1.27 | 2.22 ± 1.15 | 0.515 |
| Sigmoidal colon-Rectum | 4.39 ± 1.83 | 4.06 ± 1.73 | 0.160 |

Results are presented as mean ± standard deviation or n.
